# A rise in births following contraceptive failure in France between 2010 and 2016: results from the French national perinatal surveys

**DOI:** 10.1186/s12905-021-01255-y

**Published:** 2021-03-20

**Authors:** Camille Bonnet, Béatrice Blondel, Caroline Moreau

**Affiliations:** 1 Obstetrical, Perinatal and Pediatric Epidemiology Research Team (Epopé), Center for Epidemiology and Statistics, INSERM, Université de Paris, Maternité Port Royal, 123, Boulevard Port Royal, 75014 Paris, France; 2 Department of Population, Family and Reproductive Health, Johns Hopkins Bloomberg School of Public Health, Baltimore, MD USA; 3 Primary Care and Prevention, Centre for Research in Epidemiology and Population Health (CESP), U1018, INSERM, Villejuif, France

**Keywords:** Contraceptive behaviors, Contraceptive failure, Perinatal survey, France, Pill scare

## Abstract

**Background:**

In France, while the prevalence of contraception is high, a significant proportion of pregnancies are unintended. Following the 2012 pill scare, the contraceptive method mix, which was mostly comprised of pills and intrauterine devices (IUD), has become more diversified. In this changing landscape, our objective was to describe trends in live births resulting from contraceptive failure and evaluate how patterns of contraceptive use have contributed to observed changes between 2010 and 2016.

**Methods:**

We used data from the 2010 and the 2016 French National Perinatal surveys which included all births from all maternity units in France over a one-week period. Interviews collecting information about pre-conception contraceptive practices were conducted in the maternity ward post-delivery. Women were classified as having a contraceptive failure if they discontinued contraception because they were pregnant. Our study sample included adult women who had a live birth, had ever used contraception and did not undergo infertility treatment (n = 11,590 in 2010 and n = 9703 in 2016). We evaluated changes in contraceptive failure over time using multivariate Poisson regressions to adjust for sociodemographic characteristics and pre-pregnancy contraceptive methods.

**Results:**

Pre-pregnancy contraception evolved between 2010 and 2016 with a 12.3% point-drop in pill use, and conversely, 4.6%- and 3.2%-point increases in IUD and condom use, respectively. Use of other barrier or natural methods doubled between 2010 and 2016 but remained marginal (1.4% in 2010 vs 3.6% in 2016). Between 2010 and 2016, the proportion of live births resulting from contraceptive failure rose from 7.8 to 10.0%, with higher risks among younger, parous and socially disadvantaged mothers. The risk ratio of contraceptive failure in 2016 compared to 2010 remained higher after sociodemographic adjustments (aRR = 1.34; 95% CI; 1.23–1.47) and after adjusting for pre-pregnancy contraceptive method mix (aRR = 1.35; 95% CI; 1.25–1.49). Increases in contraceptive failures were concentrated among pill and condom users.

**Conclusions:**

Recent shifts in contraceptive behaviors in France following the 2012 pill scare may be associated with a subsequent increase in births resulting from short acting contraceptives failures.

**Supplementary Information:**

The online version contains supplementary material available at 10.1186/s12905-021-01255-y.

## Background

In France, as in many high income countries, contraceptive prevalence is high [1] but a significant proportion of pregnancies are unintended [2, 3]. Based on the last national estimates, a third of all pregnancies were unintended in 2010, with 36% ending in births [2]. As in other countries with high contraceptive prevalence, a majority of these pregnancies are due to inconsistent pill use or the use of less effective methods (condoms and other barrier or natural methods) [4–6]. After 40 years of steady decline, unintended birth rates have slightly increased in France between 2000 and 2010, due to a rise in unintended birth rates among women 30 years and older [7]. At the same time, the contraceptive landscape, which mostly relies on pill use for spacing and intrauterine devices (IUD) for limiting births, has become more diversified with the introduction of newer forms of hormonal contraception (implant, patch and ring) [8, 9]. These changes were precipitated by the French 2012 pill scare, a public response to the potential heightened risk of venous thrombosis associated with third and fourth generation pills [10]. The pill scare resulted in a substantial drop in pill use, substituted mostly by the use of condoms or other barrier or natural methods, and, to a lesser extent, by long acting reversible contraception (LARCS: IUD and implant) [8]. These changes were confirmed in the last national omnibus health Barometer survey conducted in 2016 [1]. The pill scare in France was immediately followed by a slight increase in the 2013 abortion rate, replicating the effect of the 1996 crisis in Britain which saw a rise in abortion rates [11]. However, the 2013 rise in abortion in France was short lived, as rates subsequently declined between 2014 and 2016, leveling at 14.4 per 1000 in 2017 [12–14]. While the drop in abortion rates suggests minimal impact of the pill scare on unintended pregnancy rates in France, the absence of data on unintended births limits the ability to draw such conclusions. The national perinatal surveys fill this knowledge gap by showing a 25% rise in unintended births following a contraceptive failure between 2010 and 2016 [15]. Building on these initial results, we aim to investigate how changes in pre-pregnancy contraceptive practices between 2010 and 2016 contributed to changes in the percentage of births resulting from contraceptive failures.

## Methods

### Study design and participants

This study used data from the 2010 and 2016 French National Perinatal surveys, which comprise national cross-sectional surveys conducted every five years to monitor maternal and perinatal indicators in France. Both studies were approved by the National Council on Statistical information, the French Data Protection Authority (CNIL 909003 and CNIL 2016-004) and the INSERM ethics committee. Women who agreed to participate gave their oral consent. The surveys follow a common methodology, including all live births of at least 500 g or occurring at 22 weeks of gestation or more. The surveys take place in all public and private maternity units in France during a one-week period in each survey year. Data are collected by trained midwives via face-to-face interviews with mothers in the post-partum maternity ward and by extracting information from medical records. A more detailed description of the French National Perinatal survey methodology is provided elsewhere [15].

**Fig. 1:**
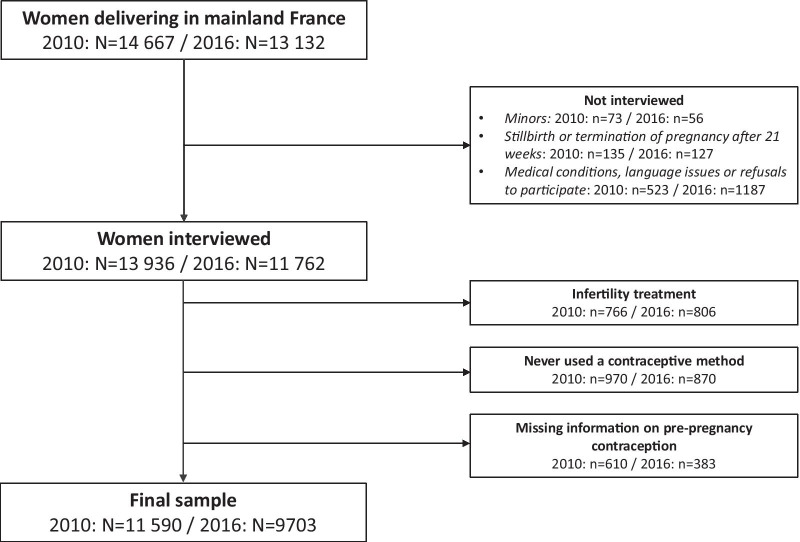
Study flow-chart

Overall, 14,667 and 13,132 women delivering a birth in Mainland France were included in 2010 and 2016, respectively (Fig. 1). Women were not interviewed if they were under the age of 18 (n = 73 in 2010 and n = 56 in 2016), or had delivered a stillbirth or had a termination of pregnancy after 21 weeks of gestation (n = 135 in 2010 and n = 127 in 2016). Another 5% of women in 2010 and 10% in 2016 were not interviewed because of their medical condition or their child’ health status, because of language issues or because they refused to participate. A total of 13,936 women in 2010 and 11,762 in 2016 were interviewed. For our analysis, we further excluded women who resorted to infertility treatment for the index pregnancy (n = 766 in 2010 and n = 806 in 2016). We also excluded women who had never used a contraceptive method (n = 970 in 2010 and n = 870 in 2016) or who had missing information regarding pre-pregnancy contraception (n = 610 in 2010 and n = 383 in 2016). Our final sample consisted of 11,590 women in 2010 and 9703 in 2016.

Women completed a 20-min face-to-face interview in which they provided information about their social background, reproductive history and contraceptive practices prior to the index pregnancy. Women described the last method used and why they had stopped using it. Based on this information, we constructed a five-category measure of the last contraceptive method used based on method mix in France and method effectiveness [16–18]. These include (1) oral contraceptive pills, (2) IUDs, (3) implants/vaginal rings or patches, (4) condoms and (5) withdrawal, periodic abstinence, or other natural methods. Vaginal rings or patches were grouped with implants as a single response option because of their very low levels of use in France. These methods were combined with the IUD in multivariate analysis, as most women in the implant/patch/ring category were likely to be implant users [1]. If two methods were used in combination, the most effective method was retained. Our primary outcome “contraceptive failure” identified women who indicated that they had stopped using contraception because they were pregnant.

We considered women’s socio-demographic information including age, cohabitation status, parity, birthplace, education, household income, and health insurance coverage at the start of pregnancy, as these characteristics are related to contraceptive practices [8, 16], unintended pregnancy [17, 18] and some factors (age, parity) affect contraceptive failure rates [19–21]. We also included abortion history as an indicator of past unintended pregnancy. Additionally, we considered women’s chronic conditions prior to pregnancy, including diabetes (insulin-dependent or non-insulin-dependent), chronic hypertension, human immunodeficiency virus (HIV), or any other chronic conditions requiring preconception care to prevent maternal or perinatal complications.

### Statistical analysis

We described the study populations interviewed in 2010 and in 2016 and explored the reasons for stopping contraception, including contraceptive failure by type of method. We conducted bivariate analysis to evaluate the association between maternal characteristics and contraceptive failure by year of survey. We examined how the effect of year of survey differed for younger and older women (< 30 and ≥ 30 years) as differential trends in unintended birth rates were reported for these age groups between 2000 and 2010 [7].We further investigated the rise in contraceptive failure in 2016 relative to 2010, accounting for changes in maternal characteristics and changes in pre-pregnancy contraceptive practices between 2010 and 2016. Because associations between maternal characteristics and contraceptive failures were similar in 2010 and 2016 (no interaction by survey year), we pooled the 2010 and 2016 datasets to evaluate the association between survey year and contraceptive failure. We conducted multivariate Poisson regressions with robust standard errors in order to compute risk ratios of contraceptive failure by survey year. First, we adjusted for maternal characteristics and then further adjusted for the type of last contraceptive method used, to assess if the observed change in method mix between 2010 and 2016 explained the association between survey year and contraceptive failure. We also conducted multivariate regression models to assess the risk ratios of contraceptive failure by survey year for each type of contraceptive method. Finally, we conducted sensitivity analyses by excluding women who stopped their contraception for other reasons than wanting a child or contraceptive failure. All statistical analyses were computed using SAS 9.4 (SAS Institute, Cary, NC, USA).

## Results

The characteristics of women delivering in French maternity services in 2010 and 2016 are displayed in Table 1. The mean age of respondents was 29.7 years in 2010 and 30.2 years in 2016 and close to 40% of participants were primiparous. About 15% of mothers were foreign born. Most women had some college education or higher (55%) while a quarter had secondary level education or less. Most respondents were covered under the mandatory national health insurance while 10% had no insurance at the beginning of pregnancy or received government insurance plan for low income or undocumented immigrants. Comparisons between survey years showed increases in maternal age and maternal education between 2010 and 2016, while other maternal characteristics remained stable.

**Table 1:** Description of study samples of women giving birth in France in 2010 and 2016

	Overall (%)	2010 (%)	2016 (%)	*P* value
		N = 11 590	N = 9703	
*Age, years*				
18–19	1.6	1.8	1.2	< 0.001
20–24	13.0	14.4	11.2	
25–29	33.4	34.0	32.7	
30–34	33.1	31.6	34.9	
35–39	15.7	15.1	16.6	
≥ 40	3.2	3.1	3.4	
*Parity*				
0	41.0	41.7	40.2	0.15
1	36.6	36.1	37.1	
2	14.9	14.6	15.1	
≥ 3	7.6	7.5	7.6	
*History of abortion*				
None	83.0	83.3	82.7	0.005
1	13.5	13.6	13.3	
2 or more	3.5	3.1	4.0	
*Country of birth*				
France	84.8	85.1	84.4	0.27
Other European country	3.4	3.2	3.6	
North Africa	5.7	5.7	5.6	
Sub-Saharan Africa	3.7	3.6	3.8	
Other country	2.5	2.4	2.6	
*Level of education*				
Middle school or less	24.6	26.3	22.5	< 0.001
High school	20.5	19.8	21.5	
Some college	21.1	22.3	19.6	
College	18.3	18.3	18.3	
Post-graduate level	15.5	13.3	18.1	
*Health insurance coverage at the beginning of pregnancy*				
National plan	87.8	88.0	87.6	0.35
Insurance for low income/undocumented or no insurance	12.2	12.0	12.4	

### Trends in contraceptive practices prior to birth between 2010 and 2016

**Fig. 2:**
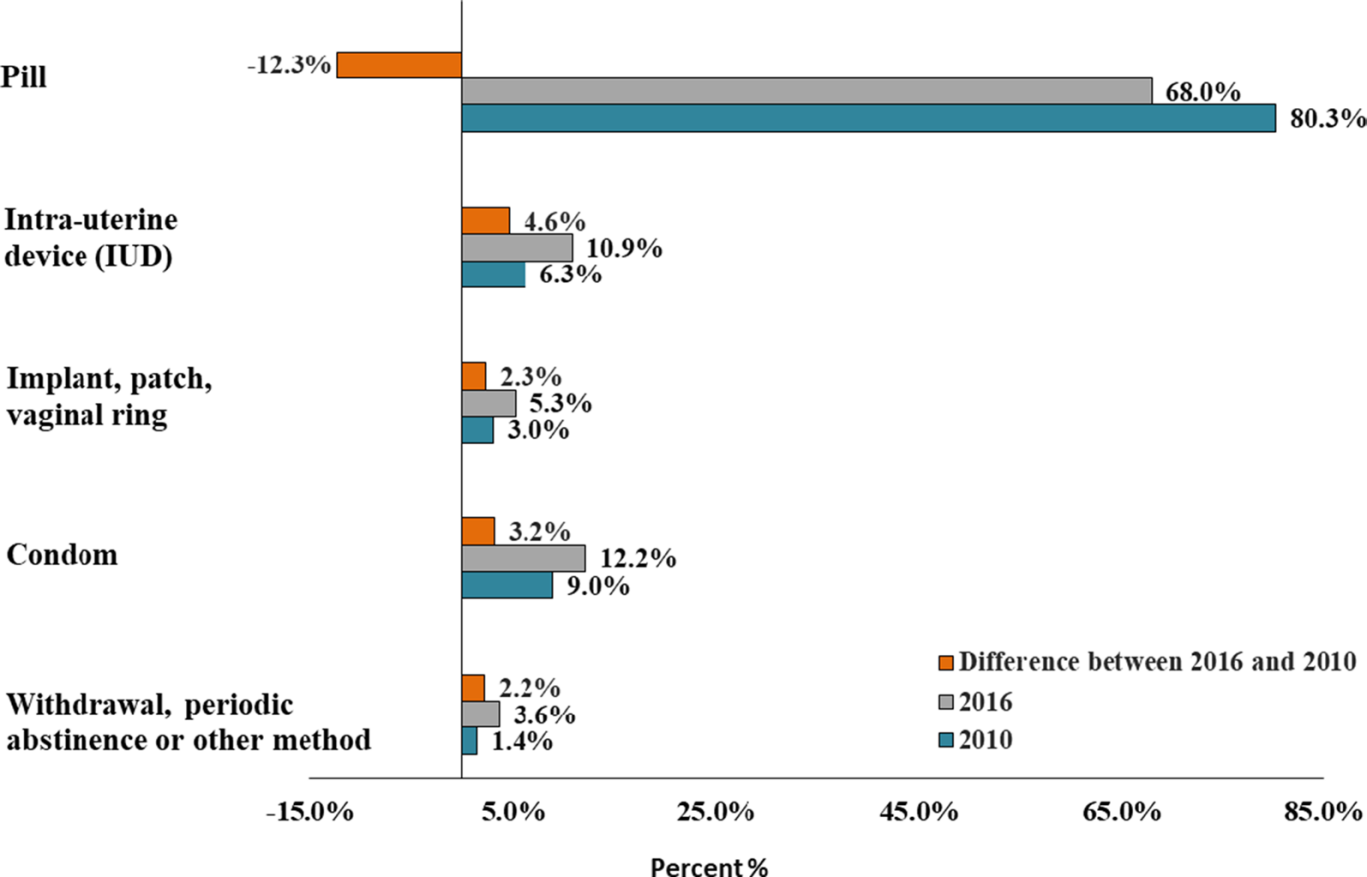
Patterns of last pre-pregnancy contraceptive method use among women who gave birth (2010 and 2016)

Contraception practices before the index pregnancy are presented in Fig. 2. The pill was the most popular method in both years, but the percentage of pill users dropped by 12.3% points between 2010 and 2016. Conversely, IUDs rose by 4.6% points reaching 10.9% in 2016 while condoms increased by 3.2% points. Use of other barrier or natural methods doubled between 2010 and 2016 but remained marginal (1.4% in 2010 vs 3.6% in 2016).

### Rise in births resulting from contraceptive failure between 2010 and 2016

In both years, a vast majority of women (79.5% in 2010 and 77.2% in 2016) stopped using contraception with the intent of becoming pregnant, another 13% discontinued contraception for other reasons, such as medical contraindication, poor tolerance or poor compliance (Table 2). A minority, but rising, percentage of women experienced a contraceptive failure (they stopped contraception because they were pregnant): 7.8% in 2010 and 10.0% in 2016. These trends were similar across age groups (less than 30 or ≥ 30 years, results not shown).

**Table 2:** Reasons for stopping the last pre-pregnancy contraception according to method type

	2010					2016					2016 versus 2010 *P* value
	No. of women	Desire to have a child N = 9216 (%)	Became pregnant N = 902 (%)	Other reason N = 1472 (%)	*P* value	No. of women	Desire to have a child N = 7493 (%)	Became pregnant N = 965 (%)	Other reason N = 1245 (%)	*P* value	
Total	11,590	79.5	7.8	12.7		9703	77.2	10.0	12.8		< 0.001
*Last contraceptive method*					< 0.001					< 0.001	
Pill	9304	80.1	7.6	12.3		6595	77.6	9.8	12.6		< 0.001
IUD	728	82.0	5.1	12.9		1059	81.1	4.7	14.2		> 0.05
Implant/patch/vaginal ring	348	73.9	4.3	21.8		517	67.5	5.0	27.5		> 0.05
Male condom	1045	76.0	10.5	13.5		1183	77.4	14.1	8.5		< 0.001
Withdrawal, periodic abstinence or other method	165	67.3	22.4	10.3		349	71.9	22.7	5.4		> 0.05

The percentage of contraceptive failures varied according to last method used (Table 2). IUDs and implants/patches/vaginal rings were associated with the lowest percentage of births following a contraceptive failure (4.7% for IUDs in 2016) while condoms and other barrier or natural methods were associated with the highest percentages of failures (14.1% for condoms and 22.7% for withdrawal or periodic abstinence in 2016). Comparisons between 2010 and 2016 show a rise in births resulting from pill failures (from 7.6 to 9.8%) and from condom use (from 10.5 to 14.1%), while births from other method failures remained unchanged (Table 2).

### Factors associated with contraceptive failures leading to birth

Bivariate analysis of maternal factors associated with births resulting from contraceptive failures are displayed in Table 3. Results indicate higher percentages of failures among younger women, women with two or more children and women with a history of abortion. Failures were also more frequent among respondents from socially disadvantaged background, including women with lower education, foreign born women and women who had no insurance or specific insurance at the beginning of pregnancy because of very low income or status as undocumented immigrant. There were no significant differences in factors related to contraceptive failures by survey year. In addition, the increases in the proportion of births resulting from contraceptive failure in 2016 relative to 2010 were consistent across sociodemographic groups.

**Table 3:** Percentage of women experiencing a contraceptive failure by women’s sociodemographic characteristics

Characteristics	Contraceptive failure					
	2010			2016		
	N	%	*P* value	N	%	*P* value
Total	11,590	7.8		9703	10.0	
*Age, years*						
18–19	212	24.5	< 0.001	117	26.5	< 0.001
20–24	1671	14.2		1090	19.4	
25–29	3943	6.1		3174	9.0	
30–34	3665	5.6		3387	7.6	
35–39	1746	7.2		1606	9.1	
≥ 40	353	10.2		329	10.0	
*Parity*						
0	4812	6.3	< 0.001	3899	8.9	< 0.001
1	4164	5.9		3598	6.5	
2	1688	10.8		1466	14.0	
≥ 3	867	19.4		737	23.9	
*Cohabiting with a partner*						
Yes	10,832	6.9	< 0.001	8932	8.7	< 0.001
No	745	21.2		757	24.8	
*Country of birth*						
France	9855	7.1	< 0.001	8186	9.1	< 0.001
Other European country	376	7.4		354	9.3	
North Africa	665	12.6		540	17.8	
Sub-Saharan Africa	416	14.9		369	15.7	
Other country	272	8.1		253	12.7	
*Level of education*						
Middle school or less	3036	12.8	< 0.001	2167	15.9	< 0.001
High school	2282	8.9		2068	11.7	
Some college	2580	6.1		1888	8.7	
College graduation	2109	4.4		1760	6.4	
Post-graduate level	1541	3.8		1746	5.4	
*Health insurance at the beginning of pregnancy*						
National plan	10,181	6.7	< 0.001	8484	8.5	< 0.001
Insurance for low income/undocumented or no insurance	1391	15.5		1206	19.9	
*Monthly household resources*						
< 1000 euros	951	17.8	< 0.001	748	21.3	< 0.001
1000–1499	1077	13.4		770	19.0	
1500–1999	1662	12.3		1191	14.2	
2000–2999	3628	6.1		2767	10.0	
3000 or more	4033	3.5		4092	4.8	
*Abortion history*						
0	9395	7.0	< 0.001	7894	9.1	< 0.001
1	1533	10.8		1269	13.6	
2 or more	356	14.0		381	16.3	
*Pre-pregnancy chronic conditions*^a^						
Yes	548	7.7	0.38	457	12.0	0.12
No	11,036	8.8		9235	9.8	

^a^Including diabetes, chronic hypertension, HIV, or any other chronic conditions requiring preconception care

Table 4 displays the results of pooled analysis of 2010 and 2016 surveys exploring changes in the risk ratio of births resulting from contraceptive failure between survey years, adjusted for maternal characteristics. Multivariate results indicate a 34% increase in the risk ratio of contraceptive failure in 2016 relative to 2010 (aRR = 1.34; 95% CI; 1.23–1.47). Results were mostly unchanged when adjusting for the last method used (aRR = 1.35; 95% CI; 1.25–1.49). Women using an IUD or implant/patch/vaginal ring prior to pregnancy were less likely to report a birth resulting from contraceptive failure compared to pill users (aRR = 0.45; 95% CI; 0.37–0.54). Conversely, condom users or women using other barrier or natural methods prior to pregnancy were more likely to describe a birth resulting from contraceptive failure compared to pill users (aRR = 1.61; 95% CI; 1.42–1.83 and aRR = 2.56; 95% CI; 2.14–3.07 respectively). Similar results were found when we excluded women who stopped their contraception for other reasons than wanting a child or contraceptive failure (see Additional file 1).

**Table 4:** Relative risk of contraceptive failure according to survey year and type of pre-pregnancy contraceptive method

	Contraceptive failure					
	Crude RR	95% CI	Model A		Model B	
			aRR^a^	95% CI	aRR^b^	95% CI
*Year*						
2010	1	Ref	1	Ref	1	Ref
2016	1.28	1.17–1.39	1.34	1.23–1.47	1.35	1.25–1.49
*Last contraceptive method used*						
Pill	–	–	–	–	1	Ref
IUD/patch/implant/vaginal ring	–	–	–	–	0.45	0.37–0.54
Male condom	–	–	–	–	1.61	1.42–1.83
Withdrawal, periodic abstinence or other method	_	_	_	_	2.56	2.14–3.07

RR: Risk ratio; aRR: Adjusted risk ratio; CI: Confidence interval

^a^Adjusted for maternal age, country of birth, parity, abortion history, level of education, live with a partner, monthly household resources and health insurance coverage at the beginning of pregnancy

^b^Adjusted for Model A covariates and last contraceptive method

Stratified analysis by the last method used suggest an increase in the risk of a birth resulting from contraceptive failure in 2016 relative to 2010, among pill users (aRR = 1.40; 95% CI; 1.26–1.56) and condom users (aRR = 1.43; 95% CI; 1.13–1.80) but no difference among IUD/implant/patch/vaginal ring users (aRR = 0.99; 95% CI; 0.69–1.43) or among women using other barrier or natural methods (aRR = 1.00; 95% CI; 0.70–1.43) (Table 5).

**Table 5:** Relative risk of contraceptive failure according to survey year stratified by last contraceptive method

	Contraceptive failure							
	Pill		IUD/patch/implant/vaginal ring		Male condom		Withdrawal, periodic abstinence or other method	
	aRR^a^	95% CI	aRR^a^	95% CI	aRR^a^	95% CI	aRR^a^	95% CI
*Year*								
2010	Ref	–	Ref	–	Ref	–	Ref	–
2016	1.40	1.26–1.56	0.99	0.69–1.43	1.43	1.13–1.80	1.00	0.70–1.43

aRR, adjusted risk ratio; CI, confidence interval

^a^Adjusted for maternal age, country of birth, parity, abortion history, level of education, live with a partner, monthly household resources and health insurance coverage at the beginning of pregnancy

## Discussion

This study suggests a rise in births following contraceptive failure between 2010 and 2016, mostly related to increases in method-specific failures rather than shifts in pre-pregnancy contraceptive method mix following the pill scare of 2012.

These results shed new light on the potential repercussions of pill scare episodes, which have been recurrent in high income countries over the last 25 years [22]. While we expected a rise in births resulting from contraceptive failure to be explained by shifts in pre-pregnancy contraceptive method mix, which mimics general population trends following the pill scare [7], our results point to an alternative explanation. Indeed, the adjustment for changes in pre-pregnancy method mix did not explain the 35% increase in risks of births following contraceptive failure between 2010 and 2016. Alternatively, we saw a rise in births resulting from pill and condom failures between 2010 and 2016, while no changes were observed among IUD users. These results suggest that population shifts in contraceptive behaviors following a pill scare, may not only affect overall coverage and method mix but also affect method-specific typical use failure rates, due to changes in user profile. While the study adjusts for a number of sociodemographic characteristics shown to affect contraceptive effectiveness, such as age and parity, other user characteristics including duration of use are not accounted for. Contraceptive failure rates are higher in the first year of use relative to longer durations of use [19, 21]. This may contribute to the observed findings that shifts in method switching following the pill scares lead to increased proportions of new users, for example in the case of condom use. This explanation, however, does not apply to pill users continuing their method after the pill scare. However, the profile of pill users may have changed following the pill scare, which may affect typical use failure rates. In any case, observed changes in method effectiveness would need to be confirmed among a representative sample of contraceptive users, as mothers who give birth are a selected group of contraceptive users who experience a failure and decide to continue the pregnancy.

An alternative explanation for the observed increase in births resulting from contraceptive failure may relate to the year 2010, which marks a peak in the French general fertility rate during the Great Recession in France (delayed compared to the US) [23]. Following job loss, some couples accelerated their childbearing timeline [23], which may have resulted in a higher proportion of intended births in 2010. Lack of information on pre-pregnancy contraception and pregnancy intentions in perinatal surveys conducted prior to 2010 prevents us from exploring this hypothesis.

The rise in unintended births resulting from contraceptive failure may also be related to a reduction in the propensity to terminate an unintended pregnancy over time. However, abortion rates were similar in 2010 and 2016. Thus, changes in abortion decisions are unlikely to explain the recent rise in births resulting from contraceptive failure.

Of note, the rise in births resulting from contraceptive failure was found to be consistent across all socio-demographic groups, sustaining social inequalities in mother’s experiences of unintended births following contraceptive failure over time.

The current study has a number of strengths including the use of large national representative samples of women who gave birth in France in 2010 and 2016, before and after the 2012 pill crisis. Women were interviewed according to the same survey instrument in 2010 and in 2016, allowing a comparison of pre-pregnancy contraceptive behaviors using comparable measures. The survey also provided ample sociodemographic measures, which were used to adjust for changes in maternal characteristics between 2010 and 2016. We therefore were able to assess whether contraceptive failures were due to changes in population composition or due to changes in contraceptive behaviors during the period of investigation, although residual maternal confounding is still possible.

While providing new insights on how shifts in contraceptive behaviors may translate in a greater proportion of births resulting from contraceptive failures, this study is not without limitations. The proportion of women who did not respond to the face-to-face interview was higher in 2016 (10.0%) than in 2010 (5.0%). This is partly due to a change in protocol which offered women the option to consent to medical record extraction but to decline survey participation in 2016. However, information on pre-pregnancy contraception was only available from questionnaire data, leading to the exclusion of women in 2016 who only consented to sharing their medical record information. Differential participation rate may affect our results, even though we adjusted for a number of maternal characteristics. Comparisons of the 2010 and 2016 samples with national data based on birth certificates indicate a slight underrepresentation of foreign mothers in 2016 (22.2% instead of 28.7% in vital statistics), most likely due to language barriers. However, there was no difference in the proportion of births resulting from contraceptive failure according to women’s country of birth, which potentially limits the effect of this sample distortion. The percentage of births resulting from IUD failures is likely over-estimated in our study, and may capture pregnancies that occurred post IUD removal, especially in the case of expulsion. We were also limited in our ability to distinguish several forms of contraception as implants, patches and vaginal rings were clustered in a single category. Results from the 2016 national Health Barometer survey suggest only 1% of women at risk of an unintended pregnancy rely on patches and vaginal rings while 4% use implants [1]. We therefore anticipate that patches and vaginal rings only account for a fraction of contraceptive failures in the perinatal surveys.

Despite these limitations, we believe our results demonstrating a rise in births resulting from contraceptive failures after the pill scare in France calls attention to potential effects of rapid changes in contraceptive user profiles on method-specific failure rates. From a provider perspective, these results emphasize the need to discuss emergency contraception during contraceptive counseling to reduce pregnancies resulting from inconsistent use of regular contraception, especially when women start new methods. For mothers who have experienced births from contraceptive failure, postpartum care is a critical opportunity to counsel about contraceptive options most suited to their needs [24, 25].

## Conclusions

This study suggests a rise in births following pill and condom failures between 2010 and 2016, following the recent shifts in contraceptive behaviors linked to the 2012 pill scare in France. In the absence of an increase in abortion rates during the same time period, these results may suggest a rise in typical use failure rates of short acting contraceptives due to changes in user profile. This hypothesis however, needs to be validated using population based data to assess contraceptive failure rates among the general population of contraceptive users. Regardless of underlying reasons for contraceptive failure, such experiences draw attention to the need for comprehensive postpartum contraceptive counseling to help women decide on the best course of action for them to prevent future unintended pregnancies and promote healthy birth spacing [24, 25].

## Supplementary Information

**Additional file 1.**. Relative risk of contraceptive failure leading to birth according to survey year and type of pre-pregnancy contraceptive method, after exclusion of women who stopped their contraception for other reasons than wanting a child of contraceptive failure (DOCX 13 KB)

## Data Availability

The datasets generated and analyzed for the current study could not be publicly available due to individual privacy of the study participants.

## Abbreviation

IUD: Intrauterine device
