# Supplementary material for: A rise in births following contraceptive failure in France between 2010 and 2016: results from the French national perinatal surveys
Source: BMC Womens Health. 2021 Mar 20;21:115. doi: 10.1186/s12905-021-01255-y (PMC7981992; doi:10.1186/s12905-021-01255-y)
Supplement: Supplementary file 1 — Additional file 1.. Relative risk of contraceptive failure leading to birth according to survey year and type of pre-pregnancy contraceptive method, after exclusion of women who stopped their contraception for other reasons than wanting a child of contraceptive failure (DOCX 13 KB) [file 12905_2021_1255_MOESM1_ESM.docx]

| **Additional file 1.** Relative risk of contraceptive failure leading to birth according to survey year and type of pre-pregnancy contraceptive method, after exclusion of women who stopped their contraception for other reasons than wanting a child of contraceptive failure | | | | | | |
| --- | --- | --- | --- | --- | --- | --- |
|  | **Contraceptive failure** | | | | | |
|  | Crude RR | 95% CI | Model A | | Model B | |
|  |  |  | aRR^a^ | 95% CI | aRR^b^ | 95% CI |
| **Year** |  |  |  |  |  |  |
| 2010 | 1 | Ref. | 1 | Ref. | 1 | Ref. |
| 2016 | 1.28 | 1.17 - 1.39 | 1.33 | 1.21 - 1.45 | 1.32 | 1.21 - 1.45 |
| **Last contraceptive method used** | | |  |  |  | |
| Pill | - | - | - | - | 1 | Ref. |
| IUD/patch/implant/vaginal ring | - | - | - | - | 0.49 | 0.40 - 0.59 |
| Male condom, withdrawal, periodic abstinence or other method | - | - | - | - | 1.74 | 1.57 - 1.94 |
| RR: Risk ratio ; aRR: Adjusted risk ratio ; CI: Confidence interval | | | | | | |
| ^a^Adjusted for maternal age, country of birth, parity, abortion history, level of education, live with a partner, monthly household resources, and health insurance coverage at the beginning of pregnancy | | | | | | |
| ^b^Adjusted for Model A covariates and last contraceptive method | | | | | | |
